# Polyamines – A New Metabolic Switch: Crosstalk With Networks Involving Senescence, Crop Improvement, and Mammalian Cancer Therapy

**DOI:** 10.3389/fpls.2019.00859

**Published:** 2019-07-03

**Authors:** Ewa Sobieszczuk-Nowicka, Ewelina Paluch-Lubawa, Autar K. Mattoo, Magdalena Arasimowicz-Jelonek, Per L. Gregersen, Andrzej Pacak

**Affiliations:** ^1^Department of Plant Physiology, Faculty of Biology, Institute of Experimental Biology, Adam Mickiewicz University in Poznań, Poznań, Poland; ^2^Sustainable Agricultural Systems Laboratory, Henry A. Wallace Beltsville Agricultural Research Center, United States Department of Agriculture, Beltsville, MD, United States; ^3^Department of Plant Ecophysiology, Faculty of Biology, Institute of Experimental Biology, Adam Mickiewicz University in Poznań, Poznań, Poland; ^4^Department of Molecular Biology and Genetics, Aarhus University, Slagelse, Denmark; ^5^Department of Gene Expression, Faculty of Biology, Institute of Molecular Biology and Biotechnology, Adam Mickiewicz University in Poznań, Poznań, Poland

**Keywords:** autophagy, cancer therapy, cell death, CRISPR/Cas9, crop improvement, polyamines-nitric oxide conjugates, senescence, transcriptome profiling

## Abstract

Polyamines (PAs) are low molecular weight organic cations comprising biogenic amines that play multiple roles in plant growth and senescence. PA metabolism was found to play a central role in metabolic and genetic reprogramming during dark-induced barley leaf senescence (DILS). Robust PA catabolism can impact the rate of senescence progression in plants. We opine that deciphering senescence-dependent polyamine-mediated multidirectional metabolic crosstalks is important to understand regulation and involvement of PAs in plant death and re-mobilization of nutrients during senescence. This will involve optimizing the use of PA biosynthesis inhibitors, robust transgenic approaches to modulate PA biosynthetic and catabolic genes, and developing novel germplasm enriched in pro- and anti-senescence traits to ensure sustained crop productivity. PA-mediated delay of senescence can extend the photosynthesis capacity, thereby increasing grain starch content in malting grains such as barley. On the other hand, accelerating the onset of senescence can lead to increases in mineral and nitrogen content in grains for animal feed. Unraveling the “polyamine metabolic switch” and delineating the roles of PAs in senescence should further our knowledge about autophagy mechanisms involved in plant senescence as well as mammalian systems. It is noteworthy that inhibitors of PA biosynthesis block cell viability in animal model systems (cell tumor lines) to control some cancers, in this instance, proliferative cancer cells were led toward cell death. Likewise, PA *conjugates* work as signal carriers for slow release of regulatory molecule nitric oxide in the targeted cells. Taken together, these and other outcomes provide examples for developing novel therapeutics for human health wellness as well as developing plant resistance/tolerance to stress stimuli.

## Introduction

Senescence is a developmental program that precedes programmed cell death (PCD) in plants and involves re-utilization/re-direction of carbon (C), nitrogen (N), phosphorus (P), and other nutrients including metals for the growth of younger leaves, seeds, and/or grain/fruit of a plant crop (Jones et al., 2012). This fundamental process has attracted investigations from different perspectives to identify the different players involved and determine if senescence process can be delayed or enhanced (Sarwat and Tuteja, 2019). For example, delaying senescence in plants induces tolerance to drought (Rivero et al., 2007). A number of plant hormones, namely, ethylene, jasmonic acid, abscisic acid, salicylic acid, strigolactones, brassinosteroids, and gibberellins are known to play an important role in plant senescence (Wojciechowska et al., 2018). Anti-senescence regulators known to impact senescence include nitric oxide (NO) (Tun et al., 2006) and polyamines (PAs) (Mattoo and Sobieszczuk-Nowicka, 2019). PAs have attracted a lot of scientific attention in recent years and evidence in favor of them being hormone-like molecules has accumulated. This review is intended to address the recent progress made in our understanding of the dynamics involved in the regulation of plant senescence by polyamines.

**Figure 1:**
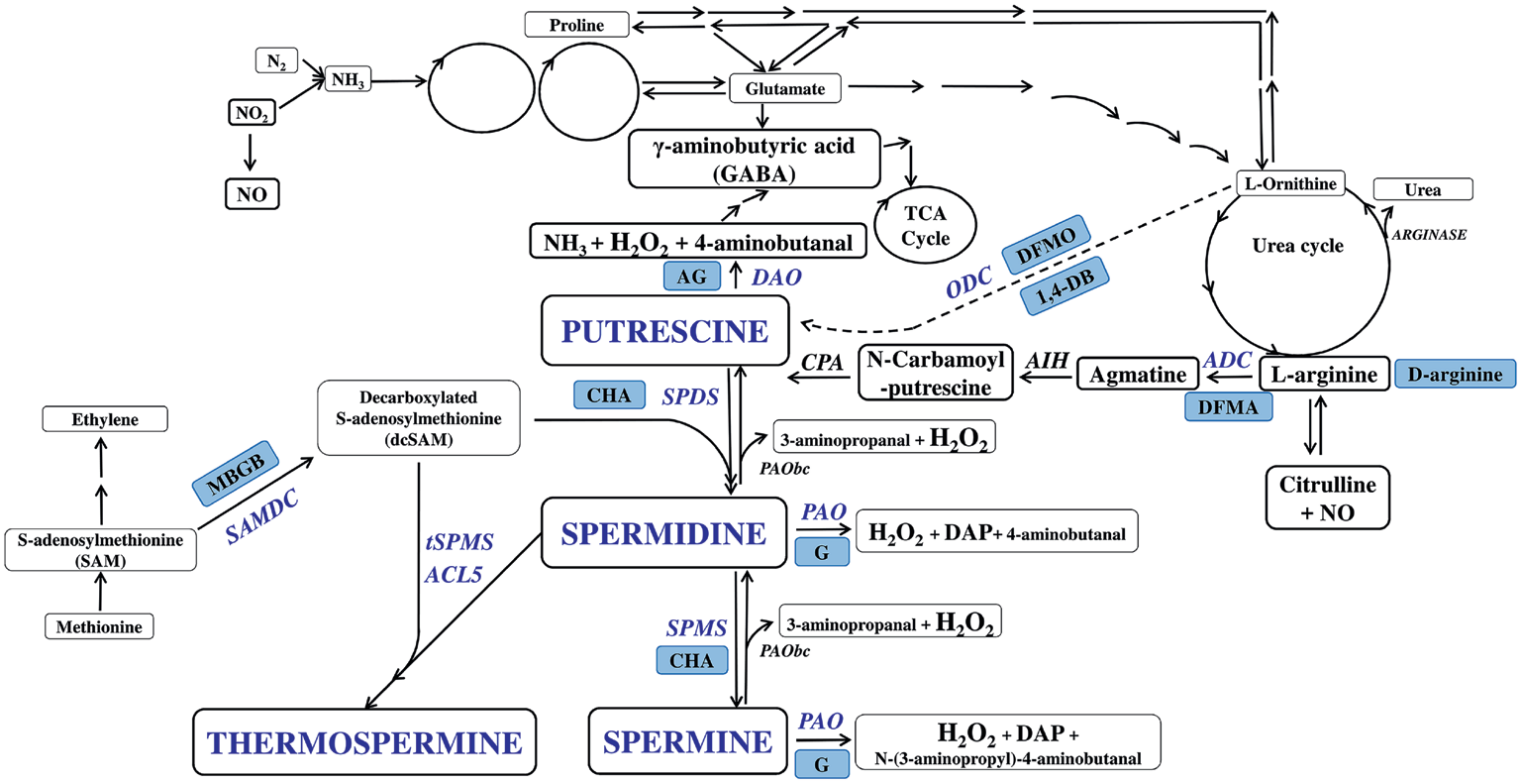
Plant polyamine metabolism pathways and its inhibition. Putrescine (Put), spermidine (Spd), and spermine (Spm) constitute major PAs that are primary amines with two or more amine groups. The biosynthesis of PAs is well established in plants. Put in many plants is synthesized from arginine (Arg) *via* agmatine catalyzed by Arg decarboxylase (ADC) and from ornithine (Orn) by Orn decarboxylase (ODC) except *Arabidopsis* in whose genome Orn decarboxylase seems not present. Put is thereafter sequentially converted to Spd and Spm/thermo-Spm (T-Spm) through successive addition of aminopropyl groups from S-adenosylmethionine catalyzed by S-adenosylmethionine decarboxylase (SAMDC). The transfer of the aminopropyl groups is catalyzed by Spd and Spm/T-Spm synthases (SPDS/SPMS/TSPMS), respectively. On the other hand, diamine (DAO) and polyamine (PAO) oxidases work in tandem to deaminate each PA, producing, in the process, the signaling molecule hydrogen peroxide (H_2_O_2_). Back-conversions from Spm to Put *via* Spd, and Spm to Spd, are catalyzed by PAOs(bc). Blue boxes indicate inhibitors of polyamine metabolism and blue font indicate polyamines and enzymes taking part in PA metabolism. ADC, arginine decarboxylase; ODC, ornithine decarboxylase; SAMDC, S-adenosylmethionine decarboxylase; SPDS/SPMS, Spd and Spm synthases; DAO, diamine oxidases; PAO, polyamine oxidases; AIH, agmatine iminohydrolase; CPA, N-carbamoyl putrescine amidohydrolase. Inhibitors: 1,4-DB – 1,4-diamino-butanone, AG – diaminoguanidine, CHA – cyclohexylammonium sulphate, D-arginine, DFMA – γ-difluoromethylarginine, DFMO – α-difluoromethylornithine, G – guazatine, MGBG -methylglyoxal*bis*-(guanylhydrazone).

The commonly used polyamines include putrescine (Put), spermidine (Spd), and spermine (Spm) (Handa et al., 2018). PA biosynthesis occurs with decarboxylation of ornithine catalyzed by ornithine decarboxylase (ODC) to form Put (Figure 1). Also, amino acid arginine is decarboxylated to agmatine by arginine decarboxylase (ADC), and agmatine in turn is converted to Put *via* N-carbamoyl-Put. Put is then successively aminopropylated to Spd and Spm by Spd synthase (SPDS) and Spm synthase (SPMS), respectively. T-Spm is the product of ACL5 (t-SPMS) in plants (Knott et al., 2007; Handa et al., 2018). The aminopropyl groups are donated by decarboxylated S-adenosylmethionine (dcSAM) whose synthesis is catalyzed by SAM decarboxylase (SAMDC; Cohen, 1998). SAMDC activity represents a common rate-limiting step in PA biosynthesis (Stanley et al., 1989; Thu-Hang et al., 2002). ODC is also a transcriptional target of the c-Myc oncogene (Bello-Fernandez et al., 1993). Unlike plants, animals possess the ODC antizyme, which control the cellular ODC levels (Matsufuji et al., 1995).

PA catabolism is mediated mainly by two classes of amine oxidases (AOs), the diamine oxidase (DAO) and PA oxidase (PAO) (Moschou et al., 2012). In arabidopsis, the AO constitutes a family of several functionally redundant genes (Tavladoraki et al., 2006; Planas-Portell et al., 2013). DAOs mainly oxidize Put and cadaverine, but also Spd and Spm with lower affinity. PAOs oxidize Spd and Spm but not Put (Angelini et al., 2010). The plant apoplastic PAOs catalyze the terminal catabolism of PAs, yielding pyrroline and 1-(3-aminopropyl)pyrrollinium from Spd and Spm, respectively, as well as 1,3-diaminopropane and H_2_O_2_ (Cohen, 1998). The plant intracellular (cytoplasmic or peroxisomal) PAOs interconvert Spm to Spd, and Spd to Put, which results also in H_2_O_2_ production (Moschou et al., 2008). Intracellular PAOs also oxidize t-Spm (Fincato et al., 2011), but the oxidation products of t-Spm have not been identified.

Animal PAOs also interconvert PAs in the peroxisomes but show preference toward the acetylated PAs produced by the inducible Spd/Spm N1-acetyltransferase (SSAT; Matsui et al., 1981; Pegg et al., 1981; Casero et al., 1991; Casero and Pegg, 1993). In animals, Spm is interconverted by cytoplasmic Spm oxidase independent of the SSAT pathway (SMO; Vujcic et al., 2002). The SMO pathway is evolutionarily conserved between animals and plants, and AtPAO1 represents the plant counterpart of SMO. The interconversion of PAs produces, in parallel to a corresponding PA, 3-aminopropanal or 3-acetamidopropanal. SMO is induced by tumor necrosis factor (TNF), resulting in the production of H_2_O_2_ which adds to inflammation, mutagenesis, and subsequently cancer development (Babbar and Casero, 2006).

Under normal conditions, AtPAO1 expression is barely detectable (Tavladoraki et al., 2006). Conditions that lead to AtPAO1 activation have not as yet been determined. In both animals and plants, genetic manipulation of PA catabolism is not an easy task, since there are several potentially redundant genes encoding AOs with similar substrate specificity. To bypass this, one alternative is to develop chemical genetics approach. Several plant DAO and PAO inhibitors have been successfully used in animals to block the catabolism and back conversion of PAs. However, their potency *in vivo* in plant biology remains largely unexplored (Moschou and Roubelakis-Angelakis, 2013).

The function of PAs as cell growth and development regulators has attracted much attention in recent years. PAs are absolutely essential for cellular viability through their role(s) in critical cellular functions, including regulation of nucleic acid and protein synthesis, and macromolecular structural integrity (Kusano and Suzuki, 2015). PA homeostasis is tightly regulated, with an excessive intracellular PAs in certain tissues leading to undesired (Wang and Casero, 2006) and desired phenotypes (Mehta et al., 2002). Thus, PAs are recognized as important ubiquitous bioactive substances with impact on several diverse biological phenomena. PAs may employ signaling mechanisms that are different than those known for the other well studied plant hormones (Mattoo and Sobieszczuk-Nowicka, 2019), since PAs are present in plant cells at higher levels, being effective often in the micro- to millimolar range (Sobieszczuk-Nowicka and Legocka, 2014; Anwar et al., 2015).

Earlier studies that suggested that PAs are anti-senescence in nature have been validated in recent studies (Sobieszczuk-Nowicka, 2017 and references therein). One such validation has come from genetic dissection of leaf senescence models, including dark-induced leaf senescence (DILS) (Sequera-Mutiozabal et al., 2016; Sobieszczuk-Nowicka et al., 2016, 2018). These developments indicate that PA catabolism plays a central role in metabolic reprogramming, directing a senescing leaf toward the programmed organ death. Thus, depending upon which direction PA metabolism undertakes, for instance whether toward synthesis/accumulation, or toward their catabolism that generates H_2_O_2_, the plant will either grow or senesce, respectively.

## DILS Versus Developmental Senescence Models

The genomic resources available for *Arabidopsis* have made it a very attractive model for the identification and functional analysis of senescence-regulated genes (Buchanan-Wollaston et al., 2003, 2005; Breeze et al., 2011). In many plants, such as barley, removal of the developing flowers and pods significantly extends the life of their leaves, while in *Arabidopsis*, male sterile mutants or plants from which the developing bolts are removed do not extend the lifespan of the leaves. Because of these differences, cereal leaves have been used over the years as a model for studying leaf development and senescence (*Zea mays* – Smart et al., 1995; *Oryza sativa* – Lee et al., 2001; *Triticum aestivum* – Uauy et al., 2006; and *Hordeum vulgare* – Kleber-Janke and Krupinska, 1997; Jukanti et al., 2008; Christiansen and Gregersen, 2014; Avila-Ospina et al., 2015; Springer et al., 2015; Wehner et al., 2015; Sobieszczuk-Nowicka et al., 2018). Distinct differences in the senescence program of *Arabidopsis* as compared to that in the monocot plants have been revealed. Senescence in cereals is generally regulated at the level of an individual leaf. Nutrients from the older leaves are remobilized for the younger leaves and eventually for the flag leaf, contributing thereby to the nutrients required for grain development. Cereal leaves have a basal meristem, the leaf tip consists of older cells, and the younger cells are concentrated at the leaf base. Such a cellular organization enables studies on senescence progression easier to differentiate (Gregersen et al., 2008). Nonetheless, the lack of coordinated development of the cells within an individual leaf introduces complexity in studying leaf senescence. Therefore, induced senescence that directs a synchronous process, such as the dark-induced senescence (DILS), has become more in vogue (Buchanan-Wollaston et al., 2005).

DILS is an extreme example of shading which induces senescence in leaves similar to that observed during normal plant development. The DILS model fits well with other important monocot crop plants, e.g., maize and rice, eliminating the confounding factors that overlap with developmental senescence such as bolting or flowering. Early and late events of leaf senescence in the DILS crop model were deciphered to reveal the time limit for dark to light transition in reversing the senescence process (Sobieszczuk-Nowicka et al., 2018). Differences in gene medleys including the hormone-activated signaling pathways, lipid catabolic processes, glutamine catabolic processes, DNA and RNA methylation and carbohydrate metabolic processes between DILS and developmental senescence processes in barley leaves have been revealed (Sobieszczuk-Nowicka et al., 2018). These studies also demonstrated that the DILS program is reversible by re-exposure of the barley plants to light prior to day-7 of dark exposure (Sobieszczuk-Nowicka et al., 2018). The senescence reversal involved regaining of photosynthesis, increase in chlorophyll and reversal of chlorophyll fluorescence vitality index (Rfd), inspite of the activation of macro-autophagy-related genes. Rfd was found to be an earliest parameter that correlated well with the cessation of photosynthesis prior to micro-autophagy symptoms, chromatin condensation, and initiation of DNA degradation.

## Polyamines and DILS Program

That applying PAs to plants prevents their senescence has been a phenomenon known for a long time (Galston et al., 1978; Kaur-Sawhney et al., 1978; Cohen et al., 1979; Apelbaum et al., 1981; Mizrahi et al., 1989; Besford et al., 1993; Legocka and Zajchert, 1999). Our understanding of plant senescence vis a vis PAs has been advanced through developments in molecular tools, genome sequencing, and genetic engineering (for instance, see Del Duca et al., 2014 and references therein; Cai et al., 2015 and references therein; Sobieszczuk-Nowicka et al., 2015, 2016; Sequera-Mutiozabal et al., 2016; Mattoo and Sobieszczuk-Nowicka, 2019). PA biosynthesis, catabolism, conjugation, interconversions, and transport all contribute to PA homeostasis (Angelini et al., 2010 and references therein; Moschou and Roubelakis-Angelakis, 2013). Transformations between individual PAs essentially contribute to darkness-induced barley leaf senescence responses (Sobieszczuk-Nowicka et al., 2016). Transcript levels and corresponding PA catabolic enzymes – DAO and PAO, increase during induced and developmental senescence making them important components of senescence-related mechanisms (Ioannidis et al., 2014; Sobieszczuk-Nowicka et al., 2016). Moreover, inhibition of PAO activity drastically slows down the senescence-associated chlorophyll loss (Sobieszczuk-Nowicka et al., 2016).

*Arabidopsis* PA back-conversion oxidase mutants have also been utilized for studying dark-induced senescence. In these mutants, conversion of Spm to Spd, and/or Spd to Put, does not occur and their senescence is delayed (Sequera-Mutiozabal et al., 2016). The delayed dark-induced senescence in these mutants was associated with accumulation of Spm levels (Sequera-Mutiozabal et al., 2016). Additionally, during this phase, these plants had a reduced production of reactive oxygen species (ROS) and, interestingly, an increase in the levels of the signaling molecule, nitric oxide (NO). These data suggest that Spm is a “signaling” metabolite, leading to protection against stress through metabolic conversions that involve ascorbate/dehydro-ascorbate redox state transitions, changes in sugar and nitrogen metabolism, cross-talk with ethylene biosynthesis, and mitochondrial electron transport chain modulation (Sequera-Mutiozabal et al., 2016). Thus, metabolic interactions between PAs, particularly Spm, occur with cellular oxidative balance and transport/biosynthesis of amino acids, likely a strategy to cope with damage during senescence.

Plants also respond to environmental factors by secreting Spd to the apoplast where its catabolism leads to H_2_O_2_ production like what is also known as a hypersensitive response. Based upon the H_2_O_2_ concentration, these cells initiate either a defense response or cell death program (Yoda et al., 2003, 2006; Marina et al., 2008; Moschou et al., 2008). It is also known that high Spd and Spm pools accumulate in the apoplast during DILS, which is associated with a gradual accumulation of apoplastic diaminopropane (PA catabolism intermediate) and H_2_O_2_ (Sobieszczuk-Nowicka et al., 2016). The basal Put levels in the apoplastic pool of PAs are an order of magnitude lower, increasing only slightly during senescence. However, Put is a dominant PA in the free PA fraction, accumulating to high levels before decreasing. The decrease in free Put is concomitant with the formation of PCA-soluble Put conjugates that accumulate at high levels in the senescing leaf, indicating that the Put-conjugating enzymes are active in a senescing cell (Sobieszczuk-Nowicka et al., 2016). Senescence-dependent remobilized nitrogen (N) and carbon (C) flow may contribute to PA conjugation, since the expression of respective protein-coding genes also increases (Sobieszczuk-Nowicka et al., 2016). That plant cells sense PAs as organic-N and stimulate turnover of N molecules has been previously substantiated and discussed (Mattoo et al., 2006, 2010).

Physiological and structural changes in barley chloroplasts during DILS occurs in association with PCA-insoluble PA conjugation, modification of chloroplast proteins, and modulation of chloroplast-localized transglutaminases (ChlTGases). TGases catalyze post-translational modification of proteins by establishing covalent linkage of ε-(γ-glutamyl) moiety on PAs (Serafini-Fracassini and Del Duca, 2008). Thus, *in situ* localization and changes in the ChlTGase activity during dark-induced senescence mirror increase in the levels of plastid membrane-bound Put and Spd (Sobieszczuk-Nowicka et al., 2009, 2015). ChlTGase catalyzes binding of [^3^H]Put and [^3^H]Spd to the photosystem proteins (Sobieszczuk-Nowicka et al., 2015). Substrates of ChlTGases in mature leaves include apoproteins of the chlorophyll a/b antenna complex, LHCII, ATP synthase and pSbS (photosystem II 22 kDa protein), proteins that are essential in energy-dependent quenching and increased thermal dissipation of excessively absorbed light energy in the photosystems (Del Duca et al., 1994; Dondini et al., 2003; Della Mea et al., 2004; Campos et al., 2010). Several stress-responsive proteins detected in the PA-bound fraction only after dark-induced senescence include the antioxidant enzyme peroxiredoxin, heat shock protein, ent-copalyldiphosphate synthase, and IAA-amino acid hydrolase (Wang et al., 2004; Van der Graaff et al., 2006; Iqbal et al., 2011; Cejudo et al., 2012). PAs in concert with TGases are functionally involved in DILS as supported by proteomic analysis and TGase activity/transcript modulation (Sobieszczuk-Nowicka et al., 2009, 2015). Thus, PAs and plant senescence do cross paths.

## Polyamines and DILS as a Model for Studying Mammalian Cancer Mechanisms

Regulation of cell death mechanisms in plants and animals (including humans) with radically different anatomy and physiology highlight PAs as universal bioregulators of this process across kingdoms (Della Mea et al., 2007; Del Duca et al., 2014; Cai et al., 2015; Handa et al., 2018). Thus, delineation of the roles of PAs should lead to a better understanding of the mechanisms in plant senescence homoeostasis causing longevity or cell death. Research into these areas should also provide new knowledge about similar mechanism(s) in mammalian systems at cellular and molecular level.

**Figure 2:**
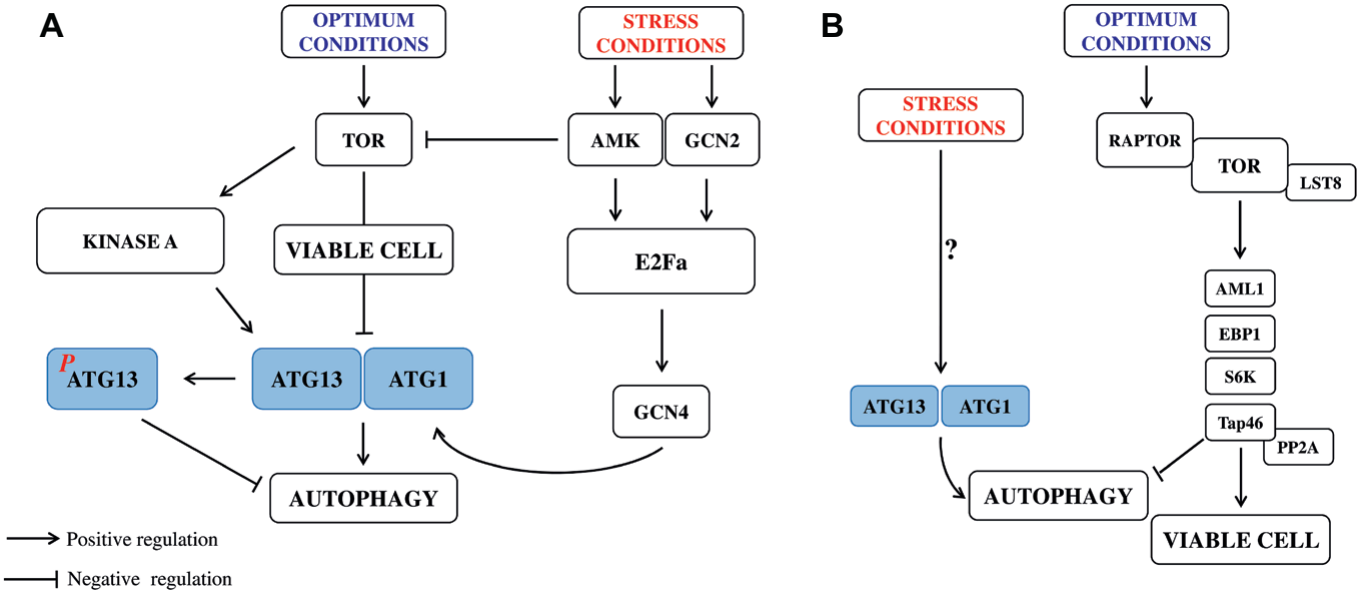
Regulation of autophagy pathways in cell development of yeast **(A)** and plants **(B)** under normal and stress conditions. **(A)** This model for the mechanism of autophagy and its regulation has been established for yeast, where the process is triggered by amino acid starvation. Multiple signaling autophagy pathways converge at the expression and activity of autophagy genes ATG1 and ATG13. Autophagy is promoted by AMP-activated protein kinase (AMPK). Conversely, autophagy is inhibited by the target of rapamycin kinase (TOR), a central cell growth regulator that integrates growth factor and nutrient signals. Under nutrient sufficiency, high TOR activity prevents ATG13 activation by phosphorylating ATG13 Ser 757 and disrupting the interaction between ATG1 and ATG13. TOR, protein kinase A, AMK (AMP activated kinase), and GCN2 (General Control Nonderepressible2) are kinases operating in autophagy signaling pathway. Elongation initiation factor 2α (E2Fa) and the transcription factor GCN4 regulate expression of ATG1 and 13. **(B)** Potential TOR signaling pathways in *Arabidopsis*. The TOR complex, including TOR, RAPTOR, and LST8 (RAPTOR recruits substrates and presents them to TOR for phosphorylation, and LST8 stabilizes the TOR complex), senses and integrates multiple upstream signals such as nutrient starvation. TOR may serve as a negative regulator of autophagy. Some TOR substrates in plants have been identified, including: AML1 (*Arabidopsis* Mei2-like1, Mei2 is a meiosis signaling molecule that has been suggested to be a potential TOR substrate, also in yeast), EBP1 (ErbB – 3 epidermal growth factor receptor binding protein), S6K (ribosomal p70 S6 kinase), and Tap46 (a regulatory subunits of PP2A (protein 2 phosphatase type 2A), which is phosphorylated by TOR, suggesting that Tap46 is a direct substrate of TOR). These substrates may function to control translation, cell growth, and autophagy (modified from Liu and Bassham, 2012).

In animal systems, longevity-promoting regimens, including the natural PAs, have been associated with apoptosis (cancer cell lines) (Madeo et al., 2010). Addition of PA inhibitors to block cell *via*bility in tumor lines and to channel cancer cells toward the path of autophagic death has been considered (Madeo et al., 2010) Longevity-promoting regimens, including caloric restriction and inhibition of TOR (Target of Rapamycin kinase signaling pathway) with rapamycin, resveratrol or the natural polyamine associated with autophagy need to be considered (Madeo et al., 2010; Zabala-Letona et al., 2017). TOR has a central role in sensing cellular nutrition and energy status and regulating cellular metabolism. It is a negative regulator of autophagy in yeast and animals (Dann and Thomas, 2006). TOR role in plants has been documented (Ren et al., 2012) following the report of its negative regulation of autophagy in *Arabidopsis* (Figure 2; Liu and Bassham, 2010). In plants, its upstream signals and downstream substrates that control the autophagy pathway still need to be investigated. A role of PAs in TOR regulation has been previously discussed (Ren et al., 2012). A model example of stress-induced selective autophagy is the C and/or N starvation. In such conditions, intensified auto-destruction allows to acquire respiratory substrates and cell survival.

**Figure 3:**
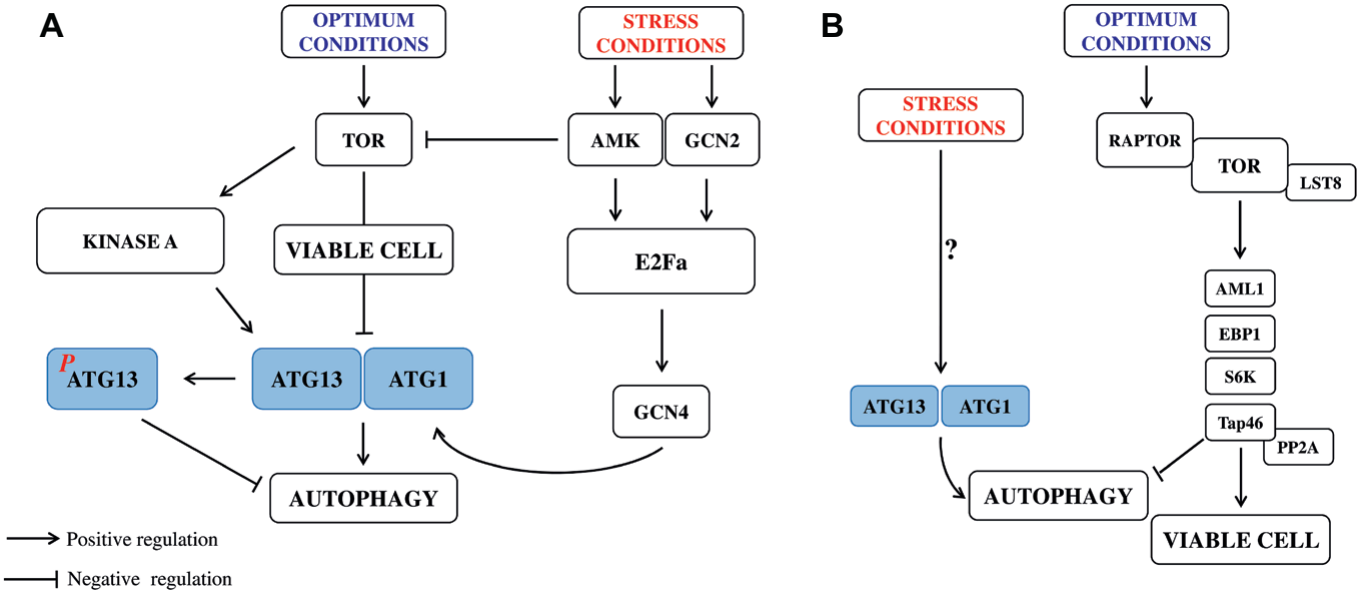
Autophagy and dark-induced leaf senescence (DILS) of barley seedlings. **(A)** Model. **(B)** Ultrastructure of autophagy symptoms of dark-induced senescing parenchyma cells. Early (days 3 and 7) and late (day 10) events of leaf senescence and time limit for reversal (arrows on the top indicate the point of no return) of the senescence process. During the initial senescence period (day 3 of darkness), tonoplast invagination, presence of small cytoplasmic fragments near or connected with tonoplast and vacuoles, and shrunken protoplasts are apparent. On day 7 of senescence, a few cells show discontinuity of the cell membrane, while by day 10, tonoplast apparently ruptures. Consequently, all the organelles undergo gradual disintegration and localized to the central part of the cell. The cell membrane increasingly loosens and, consequently, the intracellular compartmentation is lost. Cell death during senescence is distinguished by rapidly occurring changes in the chloroplasts, whereas the nucleus and mitochondria are relatively more stable, and their degradation occurs only after the final lytic stage following vacuole tonoplast rupture. In the later stages of cell death, distinguishing specific organelles is not possible. However, the shrinking of the protoplast and deformation of the cell wall are clearly observed (see the micrographs). Autophagy is apparent during ultrastructural observations of senescing parenchyma cells seen as small autophagic bodies inside vacuoles, autophagosomes presence in protoplasts, and tonoplast rupture. The changes, at each stage of DILS, are accompanied by elements of micro-, macro- and mega-autophagy. Autophagy role in the metabolic turnover of cell components as one of the mechanisms of quality control of the leaf senescence is discerned. In this process, nutrients such as carbon, nitrogen and phosphorus are released in the course of degradation of proteins, lipids, sugars and nucleic acids, and then transported to younger leaves, ripening fruits, and for seed formation. Metabolism and selective remobilization of macromolecules, which are crucial in the effective performance of the process, are accompanied first by micro- and then macro-autophagy. These studies have emphasized that the efficient regulation of autophagous process is a symptom of the vitality of senescing cells, which must at every stage maintain the ability to keep homeostasis. Bars, 200 nm days 0 and 3; 500 nm days 7 and 10 (top row of **B**); bars, 200 nm days 0, 3, 7, and 10 (bottom row of **B**) (modified from Sobieszczuk-Nowicka et al., 2018).

In mammals, autophagy is important in maintaining normal health since it prevents a number of diseases (including cancer). In plants, autophagy participates in circulation of cell components and acts as a quality control mechanism during senescence (Sobieszczuk-Nowicka et al., 2018). Therefore, DILS, may be a good model also for studying autophagy and PCD pathways because it involves defined physiological and cytological transformations: disruption of the nucleus and mitochondria, chromatin condensation, enhanced expression of cysteine proteases, autophagy proteins, and nDNA fragmentation (PCD marker) (Figure 3). It is important to note that, in spite of macro-autophagy advancement, degradation symptoms can be reversed by replacing dark conditions with light prior to the time when the process becomes irreversible. Turnover of macromolecules *via* autophagy might be critical for cell homeostasis during DILS. How the autophagy switches between cell survival and cell death is not known! Therefore, how plant cells mechanistically regulate DILS *via* autophagy is important to explore (Sobieszczuk-Nowicka et al., 2018).

PA transport as well as PA biosynthesis, degradation and conjugation play a vital role in the regulation of intracellular PA levels (Fujita and Shinozaki, 2014). PA uptake in plant cells occurs *via* energy-dependent, protein-mediated transport systems. Evidence has accumulated which suggests that paraquat, one of the most widely used herbicides, is transported by the PA transport system in diverse organisms including plants (Fujita and Shinozaki, 2014).

There is evidence that links the signaling molecule NO with PAs in living organisms. In plants, PAs overlap NO metabolism in response to developmental and stress stimuli. NO formation increases in response to exogenous PAs (Tun et al., 2006; Arasimowicz-Jelonek et al., 2009; Wimalasekera et al., 2011; Diao et al., 2017). In turn, PAs metabolism can be adjusted in a dose-dependent manner by exogenous NO (Fan et al., 2013; Filippou et al., 2013). Also, L-arginine is a precursor of both PAs and NO, and arginase has been considered as a decisive checkpoint to direct the metabolism of L-arginine to either PAs or NO (Flores et al., 2008). Also, some amino acids (e.g., methionine), which are precursors of PAs, can also affect cellular status of NO (Montilla-Bascón et al., 2017).

Generally, PA uptake is elevated in rapidly proliferating cells. Many tumor cells possess an active energy-dependent PA transport system (PTS), which selectively helps in the accumulation of endogenous PAs and structurally related compounds. Therefore, the idea of attaching cytotoxic drugs to polyamine vectors for selectively targeting cancer cells by utilizing the PTS is worth pursuing (Palmer et al., 2009). Polyamine *conjugates* have been used as signal carriers for slow release of regulatory molecules, such as NO, in targeted cancer cells (Kielbik et al., 2013). From a chemical point of view, exposing secondary amines to NO results in diazeniumdiolate formation, commonly known as “NONOates.” These compounds contain the functional group [N(O)NO]^−^ that binds an amine nucleophile adduct (Fleming et al., 2017). Numerous examples of these zwitterionic poly-amine/NO adducts are known (Hrabie et al., 1993). They are a unique group of compounds functioning in biological systems as NO donors and spontaneously release NO in aqueous solution without requiring redox or light activation (Lam et al., 2002). NONOates possess great potential in a variety of biomedical treatments, requiring rapid or prolonged release of NO *in vivo*.

Parameters, including pH, temperature, and chemical nature of the nucleophile affect the decomposition rate of NONOates, which presents a range of 1 min to 1 day under physiological conditions (Fitzhugh and Keefer, 2000). For example, spermine NONOate which is a diazenium diolate NO donor has a unique pattern of NO release (half-life around 39–73 min) which fits well with angiogenesis (Majumder et al., 2014). In general, diazeniumdiolates are useful for the study of tumor biology since they can be used as antineoplastic agents (Huerta, 2015). Also, polyamine/NO adducts were found effective in the induction of ovarian cancer cell death *via* inhibition of signal transducer and activator of transcription 3 (STAT3), serine-threonine protein kinase AKT protein phosphorylation, and downregulation of their cytocolic levels (Kielbik et al., 2013). Spermine NONOate has been adopted in plant research as an NO-releasing compound, documenting interrelationship among NO, cyclic GMP and heme oxygenase-1 in gibberellin-treated wheat aleurone layers (Wu et al., 2013).

An unusual diazeniumdiolate (*N*-nitrosohydroxylamine) was found in rhizosphere bacteria (*Paraburkholderia graminis*). *P. graminis* produces gramibactin which is a siderophore with a diazeniumdiolate ligand system (Hermenau et al., 2018). Gramibactin biosynthesis genes seem conserved in numerous plant-associated bacteria, including rice, wheat, and maize. Thus, plants could benefit not only from the iron that is mobilized by the bacteria, but also from NO released by the unique diazeniumdiolate. Although diazeniumdiolate groups are extremely scarce in nature, they can be an effective system to release NO in targeted cells. It is worth testing if such an outcome could lead to the development of novel therapeutics for human health wellness and plant resistance/tolerance to stress stimuli.

## Approaches to Polyamine Metabolism Crosstalk With the Senescence Network in Barley

Genetic mechanisms that lead to stress-induced senescence and delineate processes involved in either delaying or accelerating senescence are important to decipher. The growing world population and global climate change have necessitated the development of high yielding and nutritious crops, a theme that has become a central challenge in this century. The impact of early senescence on crop yield and quality demands that crop losses be contained since more than 30% of crop losses occur pre- and post-harvest. Modulating senescence behavior in a versatile species such as barley benefits commercial production in at least two ways: (1) delaying senescence onset and extending the photosynthetic period in malting grains can increase grain starch content; (2) accelerating the onset of senescence increases nitrogen content in grains used for animal feed.

Development of transgenic barley plants that are defective in specific PA metabolic genes can be generated *via* the ubi-overexpression, RNAi approaches or CRISPR/Cas9 (Bartlett et al., 2008; Smedley and Harwood, 2015; Holme et al., 2017). This would allow gaining “anti-aging” or “pro-aging” phenotypes as an important intervention. Loss-of-function barley transgenics deficient in PA metabolism also need to be developed. Agrobacterium-mediated transformation has been optimized in barley, conferring highly efficient transfer of foreign DNA into the barley genome within the agrobacterium T-DNA borders (Bartlett et al., 2008). Efficient over-expression of candidate genes (e.g., Holme et al., 2012) or downregulation of gene expression using RNAi techniques (e.g., Carciofi et al., 2012) in barley has been successful. CRISPR/Cas9 gene editing tools can secure efficient delivery of the Cas9 site-directed nuclease to subject the plant (barley) to genome editing (Holme et al., 2017; Lawrenson and Harwood, 2019). Notably, the inserted Cas9 and guide-RNA cassettes can be removed from selected lines after Mendelian segregation in the subsequent generations.

Senescence in barley is a complex process regulated by many diverse factors, PAs being one of them. Previously, modifying the senescence program focused on transcription factors, in particular NAC transcription factors (*No apical meristem, ATAF1/2, cup-shaped cotyledon 2*), which are known key regulators of plant senescence (e.g., Christiansen and Gregersen, 2014). Thus, plants constitutively over-expressing the senescence-associated NAC gene HvNAC005 showed an early senescence, albeit with stunted plants (Christiansen et al., 2016). In order to manipulate the PA metabolism, there is a range of potential candidate genes, e.g., DAO and PAO, that could be downregulated by targeted knockout using the CRISPR/Cas9 mutation strategy. Designing a strategy to systematically knockout the genes involved in the biosynthesis, degradation and sequestering of PAs could provide an array of plants with different senescence phenotypes. Studies with these lines could help delineate the role(s) of different genes in the homeostasis of PAs in a plant. In order to have plants that can be grown without restrictions under field conditions, one possible alternative is to select promising CRISPR/Cas9 mutations and mimic them by TILLING screening of a classical mutant collection.

Constitutive or inducible overexpression of PA biosynthetic genes (ADC, ODC, SAMDC, SPDS, and SPMS) from different plant and animal sources in *Arabidopsis*, rice, tobacco, tomato, eggplant, and pear have resulted in increasing the endogenous levels of a known PA. This led to enhanced plant tolerance to various abiotic stresses, such as salt, drought, low/high temperature, wounding, ozone, flooding, heavy metals (Cu, Cr, Fe, and Ni), acid stress, and oxidative stresses (Hussain et al., 2011; Moschou et al., 2012; Tavladoraki et al., 2012; Shi et al., 2013). Conversely, knockout mutants of AtADC1/2 with lowered level of Put had decreased tolerance to salt and freezing (Cuevas et al., 2008). Also, knockout mutants of AtSPMS/AtACL5 exhibited less Spm accumulation and had decreased tolerance to salt, drought, and heat stresses (Tavladoraki et al., 2012).

A number of studies have also utilized the inhibitors of PA biosynthesis or catabolism. Some chemicals were identified as inhibitors of PA metabolism (Kaur-Sawhney et al., 2003). Among these, difluoromethylarginine (DFMA) and difluoromethylornithine (DFMO) are reversible inhibitors of ADC and ODC, respectively; methylglyoxal-bis guanylhydrazone (MGBG) is a potent inhibitor of SAMDC; however, it can also inhibit ADC and PAO activity; 1,4-diamino-butanone (1,4-DB) is a Put inhibitor; cyclohexylamine (CHA) is a competitive inhibitor of SPDS; D-arginine is also a PA biosynthetic inhibitor, though less compatible form of arginine compared with L-arginine (Kaur-Sawhney et al., 2003). Guazatine blocks action of PAO, while aminoguanidine inhibits DAO (Figure 1). These inhibitors have unspecific roles in PA metabolism. Thus, using them requires caution, particularly when interpreting results obtained from such a pharmacological approach.

Examples of the use of PA supplementation and PA metabolism’s blockers in senescence models and the response of these systems to PA metabolism changes have been discussed (Cai et al., 2015; Sobieszczuk-Nowicka, 2017). Generally, it has been considered that the supplementation of exogenous PA or blocking their oxidation has an anti-aging effect, while PA biosynthesis inhibitors that block cell viability in animal model systems (cell tumor lines) to prevent cancers have also been tested (Russell and Snyder, 1968; Upp et al., 1988; Manni et al., 1995; Wallace and Caslake, 2001; Gilmour, 2007; Nowotarski et al., 2013). PAs as promoters of cellular proliferation and growth became a consideration after ODC activity was detected in regenerating rat liver, chick embryo, and various tumors (Russell and Snyder, 1968). The higher levels of PAs in cancerous cells suggest their possible role in tumor formation/growth (Upp et al., 1988; Manni et al., 1995; Wallace and Caslake, 2001; Gilmour, 2007). This rationale advanced the use of an inhibitor of ODC, difluoromethylornithine (DFMO), as a chemopreventive agent to treat cancerous cells (Nowotarski et al., 2013).

## Conclusions

**Figure 4:**
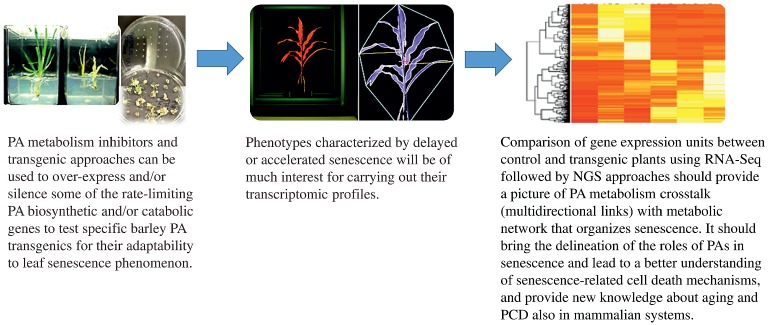
Polyamine metabolism crosstalks (multidirectional links) with the metabolic network during the induced-senescence process. Polyamines as a metabolic switch for barley leaf senescence is a good model for developing a molecular basis of the process in order to apply such information for developing resilient crops for the future. PA metabolism inhibitors as well as transgenic approaches can be used to over-express and/or silence some of the rate-limiting PA biosynthetic and catabolic genes to test specific barley PA transgenics for their adaptability to leaf senescence phenomenon. PAs may, in future, play a role in reprogramming plant senescence that can be altered for a pro-growth phenotype by exogenously directed application of natural and synthetic PAs. This can help plants to develop tolerance to the broad spectrum of stress factors and thereby lead to higher plant productivity. The delineation of the roles of PAs in senescence should lead also to a better understanding of senescence-related cell death mechanisms and provide new knowledge about PCD in mammalian systems since PAs are universal bioregulators of these processes across kingdoms.

All types of stresses, including darkness, limit plant growth and crop productivity. This has more dire consequences in view of the fact that, based on FAO data, the world will need 70% more food to feed the anticipated 9 billion people by 2050. Thus, achieving global food security while reconciling demands of the environment is the greatest challenge faced today by mankind. Thus, it is importantly clear that increasing plant productivity, improving food quality and enhancing agricultural sustainability can no longer be ignored. In this regard, research on the anabolism or catabolism of polyamines plays an important role in reprogramming metabolic switches such that plant leaf senescence can be altered for a particular pro-growth phenotype or where organ death is enhanced without affecting the energy-use efficiency of plants. Likewise, exogenous application of natural or synthetic PAs can help plants to improve their tolerance against a broad spectrum of stress factors which, in turn, should lead to higher plant productivity as well as extend the boundaries of crop cultivation. Details on PA signaling transduction pathway(s) and the crosstalk between PA and other plant regulators/hormones are basically unknown and just beginning to be unraveled. Scientists delving in the PA science arena are already making inroads into PA omics profiling, developing novel germplasm by genetic engineering, and unraveling the interactions between PAs, other hormones and stress-responsive molecules such as NO. Such studies should bring new insights to our understanding of PA-related stress induced-senescence and cell death mechanism(s). Comparison of gene expression units between control and transgenic plants using RNA-Seq followed by NGS (Next Generation Sequencing) approaches should provide a broader picture of interconnected gene medleys that lead to senescence and other mechanisms regulated by PAs (Figure 4). The delineation of the roles of PAs in senescence should lead to a better understanding of senescence-related cell death mechanisms and provide new knowledge about senescence and PCD also in mammalian systems at cellular and molecular level since PAs are universal bioregulators of these processes across kingdoms. Such an outcome should also contribute in developing new strategies to be applied toward human health wellness.

## Data Availability

All datasets for this study are included in the manuscript and/or the supplementary files.

## Author Contributions

All authors listed have made a substantial, direct and intellectual contribution to the work, and approved it for publication.

### Conflict of Interest Statement

The authors declare that the research was conducted in the absence of any commercial or financial relationships that could be construed as a potential conflict of interest.

## Abbreviations

ADC: Arg decarboxylase
Arg: Arginine
ACC: 1-aminocyclopropane-1-carboxylate
C: Carbon
DAOs: Diamine oxidases
DILS: Dark-induced leaf senescence
N: Nitrogen
NO: Nitric oxide
NONOates: Exposing secondary amines to high pressure of NO results in diazeniumdiolates formation commonly known as “NONOates”
Orn: Ornithine
ODC: Orn decarboxylase
PAs: Polyamines
PAOs: Polyamine oxidases
PAOs(bc): Polyamine back-conversions oxidases
Put: Putrescine
PTS: Polyamine transport system
Rfd: Chl fluorescence decrease ratio called vitality index Rfd = (Fm − Fs)/Fs
Spd: Spermidine
Spm: Spermine
SAM: S-adenosylmethionine
SAMDC: SAM decarboxylase
TOR: Target of Rapamycin kinase signaling pathway
